# Using position rather than color at the traffic light – Covariation learning-based deviation from instructions in attention deficit/hyperactivity disorder

**DOI:** 10.3389/fpsyg.2022.967467

**Published:** 2022-09-07

**Authors:** Robert Gaschler, Beate Elisabeth Ditsche-Klein, Michael Kriechbaumer, Christine Blech, Dorit Wenke

**Affiliations:** ^1^Department of Psychology, FernUniversität in Hagen, Hagen, Germany; ^2^Department of Psychology, Universität Koblenz-Landau, Landau, Germany; ^3^Department of Psychology, Private University of Applied Sciences, Göttingen, Germany

**Keywords:** ADHD, instructed task set, covariation learning, strategy change, incidental learning

## Abstract

Based on instructions people can form task representations that shield relevant from seemingly irrelevant information. It has been documented that instructions can tie people to a particular way of performing a task despite that in principle a more efficient way could be learned and used. Since task shielding can lead to persistence of inefficient variants of task performance, it is relevant to test whether individuals with attention deficit/hyperactivity disorder (ADHD) – characterized by less task shielding – are more likely and quicker to escape a suboptimal instructed variant of performing a task. The paradigm used in this online study builds on the observation that in many environments different covarying features could be used to determine the appropriate response. For instance, as they approach a traffic light, drivers and pedestrians monitor the color (instructed stimulus feature) and/or the position of the signal (covarying stimulus feature, more efficient in case of reduced color sight). Similarly, we instructed participants to respond to the color of a stimulus without mentioning that color covaried with the position of the stimulus. In order to assess whether with practice participants would use the non-instructed feature position to an increasing extent, we compared reaction times and error rates for standard trials to trials in which color was either ambiguous or did not match the usual covariation. Results showed that the covariation learning task can be administered online to adult participants with and without ADHD. Performance differences suggested that with practice ADHD participants (*n* = 43 out of a total *N* = 245) might increase attention to non-instructed stimulus features. Yet, they used the non-instructed covarying stimulus feature to a similar extent as other participants. Together the results suggest that participants with ADHD do not lag behind in abandoning instructed task processing in favor of a learned alternative strategy.

## Introduction

Traffic lights are but one example of everyday tasks in which people can select a response based on the stimulus feature mentioned in the instruction or use an alternative stimulus feature which correlates with the instructed one. While we have been instructed to use the color of the light to select a response, we could alternatively or in addition use the position of the light. This is because red is placed in the upper part of the traffic light. For instance, Overton and Brown (1957) reported that people who were not aware of their reduced color vision could use the position of the light in a compensatory manner. It is less clear what determines whether with practice people employ a non-instructed correlated stimulus feature if the instructed stimulus feature remains available. While often changes in cognitive control have been linked to cognitive conflict between instruction-based task processing and alternative response tendencies (e.g., Botvinick et al., 2001), less is known about whether and how people deviate from performing a task as instructed to employing correlated stimulus features.

Adaptive behavior in changing environments necessitates balancing stability and flexibility (cf. Hommel, 2015; Mekern et al., 2019). Stability is needed to shield successful behavior against competing and distracting influences. Flexibility is needed to adapt to changing demands and to establish new habits. Resources are either allocated for sustaining the current task representation or for exploring novel structures in the environment that might turn out to be useful. This trade-off has been framed as stability-flexibility paradox (Goschke, 2000) or exploitation-exploration problem (e.g., Daw et al., 2006). Task sets play an important role in stabilizing goal directed behavior. They allow task control by parameters that determine processes of stimulus identification, response selection and response execution (Vandierendonck et al., 2010). Features of stimuli and responses are being weighted based on usefulness to secure goal-directed behavior (e.g., Hommel et al., 2001). Task sets are either established as a result of feedback-based learning (Nakahara et al., 2002) or based on verbal instructions (Logan and Gordon, 2001; Wenke and Frensch, 2005). Once established, task sets stabilize goal-directed behavior by shielding from irrelevant information to gain attention (e.g., Dreisbach and Haider, 2009; Dreisbach and Wenke, 2011). While task sets allow stable goal pursuit in stable environments, they can hinder learning about alternative ways to deal with a task (Kamin, 1968; Doll et al., 2009). The shielding function of task sets can lead to rigidity such as documented in the set effect (e.g., Luchins, 1942; Ninomiya et al., 2022).

Instructions can determine how we handle a task (e.g., Hommel, 1993; Wenke and Frensch, 2005; Dreisbach and Haider, 2008, 2009; Sakai, 2008; Meiran et al., 2015). Yet, the literature on skill acquisition shows that practice improves task performance (e.g., Logan, 1988, 1992; Heathcote et al., 2000; Roeder and Ashby, 2016). Both factors – *learning* (e.g., Fan and Turk-Browne, 2016; Qian et al., 2016) as well as *instructions* (cf. Hommel, 1993; Wenke and Frensch, 2005) – are thought to affect attentional control during task execution. Attentional control in turn secures that the processing of task-relevant information is prioritized over the processing of information that has proven to be, or was instructed to be, less relevant (cf. Miller and Cohen, 2001). Previous research has shown that the concurrent influence of learning and instructions on attentional control can lead to interference, for example when instructions hinder learning of contingencies in the task material (Doll et al., 2009; Schmidt and De Houwer, 2012; Bocanegra and Hommel, 2014). While instructions can shield processing of relevant information from influences of irrelevant information (Dreisbach and Haider, 2008, 2009), this does not rule out that participants can learn about an irrelevant feature that provides an alternative strategy for performing a task (Tsushima et al., 2008).

The shielding function of task sets does not seem to be perfect. Information that originally was deemed irrelevant can be acquired and used for performing a task (e.g., Tsushima et al., 2008). Changes in reward contingencies (e.g., Daw et al., 2006), reward prospects (i.e., Fischer et al., 2018; Fröber et al., 2020; Fröber and Dreisbach, 2021) and response conflict (e.g., Botvinick et al., 2001; Botvinick, 2007) can influence the balance between stability and flexibility. Less is known about the reduction of task shielding when instructed stimulus-response mappings remain valid and there is neither response conflict nor a change in reward contingency. This is surprising as people face correlated stimulus- and response features in many parts of their daily environment. One approach to target this issue is to compare groups of persons potentially differing in task shielding. For instance, referring to differences in prefrontal cortex maturation among children and adolescents, Schuck et al. (2022) tested for differences among children and young adults to deviate from instruction-based task performance. Children and young adults to a similar instead engaged in task-performance controlled by a stimulus feature that was not mentioned in the instructions, but was correlated with the instructed stimulus feature.

In the current research we explored whether adults with and without *attention deficit/hyperactivity disorder* (ADHD) differ in the extent to which instructions determine their task processing. In particular, we were interested in how with practice they could escape from the power of instructions to control task processing (cf. Colzato et al., 2022). The power of the human capability to configure task processing based on instructions has long been acknowledged. For instance, reporting on animal experiments, Tolman (1948) pointed out that humans obtain and use a somewhat unfair starting advantage in coping with a new task: they are usually told upfront in the instructions which features of the situation are relevant and which response options are at stake. Given the instruction-based head-start, humans are not bothered with having to find out what might be relevant in a given situation. Yet, this head-start might come at the price that correlated stimulus features that offer alternative ways of performing the task might be neglected. This price might differ for people with vs. without ADHD.

Underperformance and undesired behaviors have often been reported to be associated with ADHD (cf. Loe and Feldman, 2007; Daley and Birchwood, 2010; Luo et al., 2019; Hoogman et al., 2020) and so have been negative stereotyping and stigmatization (Brandau et al., 2007; Lebowitz, 2016). Contrasting with this negative perspective, individuals with ADHD might play out particular strengths in some domains and tasks (cf. Lesch, 2018). ADHD has been associated with traits and processes that might support the escape from instruction-based task shielding and support the discovery and usage of alternative correlated stimulus features for task performance. ADHD has been discussed with perspective on the research on mind wandering (cf. Bozhilova et al., 2018) and creativity (Hoogman et al., 2020). Some authors suggest that coming up with novel and useful ideas is a positive aspect and strength associated with ADHD (Hoogman et al., 2020). Creativity has been linked to increased impulsivity and distractibility which are also ADHD symptoms (Zaragoza, 2010; Zabelina et al., 2016). Creativity can show in more flexible switching of attention and perspectives in problem solving (cf. Nijstad et al., 2010; Zhang et al., 2020). This flexibility might be fostered by diversion of attention found in ADHD (cf. White and Shah, 2016, Boot et al., 2017). Hoogman et al. (2020), see also Kasof (1997) and Zabelina et al. (2016) suggest that reduced shielding of the instructed task set might result in a higher likelihood that stimulus features that are not part of the instructions enter working memory together with instructed stimulus features. This in turn might help to learn that the non-instructed stimulus feature might be used for response selection. Selective attention to stimulus features carrying an unannounced regularity in the task material has been characterized as a prerequisite for implicit learning processes that lead to the acquisition of knowledge about the redundancy in the task material (Frensch and Miner, 1994; Jiménez and Méndez, 1999; Hoffmann and Sebald, 2005; Gaschler et al., 2012).

Summarizing studies on potential benefits in creativity for people with ADHD, Hoogman et al. (2020) reported that increased divergent thinking was present in people with high (subclinical) ADHD scores, but no benefit was observed in individuals with the disorder. The authors suggested that only for the former, the benefits of diversion of attention might outweigh the drawbacks of less stable task processing. We suggest that this might shift when the alternative variant of task processing is simple and straightforward. In the current study, participants who break instruction-based task shielding and attend stimulus features that are not mentioned in the instructions can discover a simple alternative for response selection.

**FIGURE 1:**
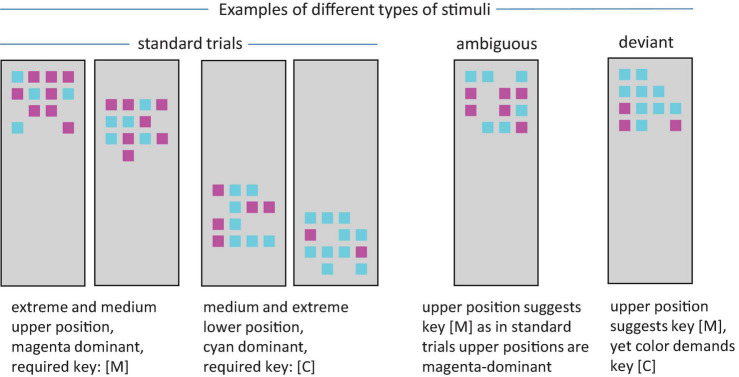
Examples of stimuli varying stimulus position and ratio of cyan and magenta squares. In standard trials, dominantly magenta arrays were always placed in the upper part of the reference frame and dominantly cyan stimuli always in the lower part. For deviant trials, the color-position relationship is reverse. For ambiguous trials, there is no dominant color.

In the current work we explored whether adults with and without ADHD could perform an incidental covariation learning task and would differ in using the non-instructed correlated stimulus feature for response selection. We developed a task in analogy to the traffic light situation. It should allow us to study the deviation from initial instruction-based processing (use color to select a response) to alternative processing (use the covarying stimulus feature position). As detailed below, people were instructed to respond to the dominant color in an array of color squares placed in a reference frame. To avoid that participants would be reminded of a traffic light, we used magenta and cyan (rather than red and green). Yet, similar to the traffic light situation, there was a redundancy. On each trial participants were presented with an array of squares of cyan and magenta (see Figure 1). They were instructed to respond by keypress to the color that was more frequent in the array. Importantly, throughout practice any stimulus in which magenta dominated was positioned in the upper part of the reference frame (while it was positioned in the lower part in case cyan dominated). We developed this setup based on earlier work on covariation-based strategy change with a color-position covariation (Schuck et al., 2015, 2022; Gaschler et al., 2019). More closely resembling the traffic light setup, in the current work color was the instructed stimulus feature while position was the covarying feature. Switching the roles of color and position compared to earlier work yielded the benefit that a reaction time (RT) based measure of processing of the non-instructed stimulus feature became available. As detailed below we could track throughout practice how the non-instructed covarying stimulus feature increasingly influenced task processing.

As suggested above, participants with and without ADHD might differ in how likely and quickly they incorporate the non-instructed feature stimulus position into task processing. We explored this issue in a setup where in principle throughout practice either of the features could have been used for response selection. Yet, there is a second aspect to consider when studying how people abandon instruction-based task processing. People with and without ADHD might differ in how quickly they re-establish instruction-based task processing when, in a test block, the instructed and the learned variant are put into conflict. Past work on strategy change has documented that practice with material with redundant features can blind participants to detect cognitive conflict once it occurs (cf. Woltz et al., 2000). Hence, we arranged the setup so that in a test phase the learned covariation between position and color was broken in some of the trials. This allowed us to observe whether learners stick to the learned short-cut strategy or obeyed the original instructions. While the position of the colors in traffic lights should not be changed due to safety concerns, such a test is informative in our setup. When breaking the color-position covariation in some trials in the test phase, participants might in part provide a position-based response rather than sticking to the instructions and using color. Additionally, when managing to obey to the instructions in these trials, participants might be slowed in responding, because they have to resolve the conflict between the color-based and the position-based response tendency (cf. Botvinick et al., 2001).

## Materials and methods

### Participants

The online study programmed in lab.js (Henninger et al., 2022) explicitly addressed adult participants with and without ADHD who participated on a voluntary basis. The German Self Support Group ADHD (Selbsthilfegruppe ADHS Deutschland e.V.) supported recruitment by distributing the call. In addition the study was advertised to students of FernUniversität in Hagen. At the state-run German distance teaching university, most students are working and studying part-time and characterized by large heterogeneity in age (cf. Stürmer et al., 2018). Participants were asked whether they had been diagnosed with ADHD. This report was validated based on the six-item Adult-ADHD-Self-Report-Scale (Kessler et al., 2005).

Ten of all participants had to be excluded because they did not fully complete the experimental task, 18 had to be excluded because they had an error rate higher than 25% in standard trials. This left us with a sample of 245 participants. Of these, 43 reported that they had been diagnosed with ADHD. The latter affirmed to a higher number of symptoms in the six-item Adult-ADHD-Self-Report-Scale (*M* = 4.48, SD = 1.26) compared to the other participants [*M* = 1.79, SD = 1.6; *t*(243) = 10.39, *p* < 0.001]. ADHD (*M* = 35.57 years, SD = 11.99) and non-ADHD participants (*M* = 35.13 years, SD = 13.04) did not differ in age [*t*(173) = 0.43; *p* = 0.866]. Note that some reports on demographical data were lost due to mistakes in adhering to the format of data entry or due to skipping this question. Of the ADHD participants, 21 were female and 21 were male and one selected diverse. Of the non-ADHD participants, 144 were female and 58 were male.

### Design, task, and procedure

We obtained a positive vote of the ethics committee of the department of psychology of Humboldt-Universität, Berlin (former affiliation of Robert Gaschler and Dorit Wenke). Participants were tested after giving their informed consent. While Group (ADHD vs. non-ADHD) was a between subjects factor, other factors such as Block, Color Ratio, and Position Extremeness were within-subjects manipulations.

Before instructions, participants indicated that they were able to differentiate colors. Participants were instructed to respond manually to the color of an array of colored squares (Figure 1). In the 4 × 4 matrix, 12 positions were filled with small color squares of either magenta or cyan. Participants were asked to press the M-key if there were more squares of magenta and the C-key if there were more squares in cyan with the index fingers of their left and right hand. They were told to respond as quickly as possible to the stimuli presented only briefly and to guess a response if they were not sure which color was more frequent. Furthermore, the instructions mentioned that the color array would be presented in a rectangular frame at a position varying from trial to trial. Yet, it was not mentioned that the position would be correlated with the dominant color.

Each trial started with the presentation of the reference frame. After 500 ms, the stimulus was presented for 500 ms and erased afterward. Hence, there was no time to count the color squares. A quick estimation was required instead. When the participant responded, the reference frame (and the stimulus in case it was still present) were erased. The 1,000 ms before the beginning of the next trial were used for error feedback which was only applied in standard trials with incorrect response: In this case, “Fehler” (German for error) was presented centrally and in bold red letters. If a participant would not respond within 3000 ms, there was written feedback that responding was too slow.

#### Block and color ratio

The task consisted of the Training Blocks 1 to 3 and the Test Block 4, not being announced as a test. Each of the training blocks consisted of 100 trials. In 80 of the trials one of the colors was dominant. There were 10 trials for each of the following color ratios: 2:10, 10:2, 3:9, 9:3, 4:8, 8:4, 7:5, and 5:7. In the remaining 20 trials, both colors were equally frequent (6:6). Hence, these trials were ambiguous with respect to the response required based on the instructed color-key mapping. Due to the variation of the color ratio in the standard trials and the short presentation time (see below), the stimuli in ambiguous trials should not be experienced as outliers, but as stimuli that are difficult to discriminate based on the instructed feature color (cf. Gaschler and Nattkemper, 2012). With practice the unannounced covariation between color and position might be used to determine the response in the ambiguous trials: For the 80 standard trials per block, stimulus position could be used in principle as an alternative source to determine the response. If the stimulus was dominantly magenta, then it was always presented in the upper part of the reference frame. If it was dominantly cyan, it was always presented in the lower part. Hence the mapping was similar to a traffic light where red is on top of green, so that people could in principle use light position alternatively or in addition to the instructed feature color to determine the appropriate response.

Block 4 also contained 100 trials and served as an unannounced test block. Apart from two different variants of test trials it contained 64 standard trials. On the one hand, there were again 20 ambiguous trials. We were interested to assess to what extent people would use the non-instructed stimulus feature position, if the instructed feature color could not be used to determine a response. On the other hand, there were 16 deviant trials in which the color-position covariation was broken: If magenta dominated in a stimulus, the stimulus was presented at a lower position. If cyan dominated, it was presented in an upper position. We were interested to what extent people would base their response on the non-instructed (but learned) feature position instead of the instructed stimulus feature color, if these features were in conflict. To evaluate position usage in deviant trials, the error rate in standard trials should be used as a baseline. If the error rate in deviant trials and standard trials of Block 4 is identical, one would conclude that position is not driving response selection in deviant trials. Yet, to the extent that the error rate is higher in deviant as compared to standard trials, erroneous deviant trial responses can be attributed to the impact of the formerly covarying stimulus feature position (rather than other processes leading to errors).

In addition we wanted to assess to what extent people would be slowed down by successfully resolving the conflict between instructed and learned stimulus feature. To this end we tested whether there was a slowing in deviant as compared to standard trials by comparing average RTs to deviant trials with instruction-congruent responses and RTs to standard trials.

#### Position extremeness

Throughout all variants of trials and in all blocks, stimuli were always presented in the upper or the lower half of the reference frame. Yet, the stimulus could either be placed at the border of the reference frame (extreme upper or lower position) or right below/above the imaginary mid line of the reference frame (medium high/low). In any case, position would have been clearly discriminable for an observer attending to this feature. The two variants of higher and two variants of lower positions were used with equal frequency across all types of trials in all blocks. We included the position variation in order to obtain an index of position processing that would be available even in standard trials. We assumed that the RT of stimuli in extreme vs. medium position would differ more if position was attended.

After completing the experimental task, participants were explained that in part stimulus color and position had covaried and that we were interested to learn to what extent they have become aware of this. Participants were asked about whether they had (1) noticed that some color-position combinations had been more frequent than others, and (2) whether they had used this knowledge to improve task performance (binary response format in either case). Last, they were asked to indicate which of the two colors was more frequent if the stimulus was placed in the upper part of frame. Finally, participants were fully debriefed about the research question and offered a contact e-mail in order to ask questions if they wished.

## Hypotheses

We expected that ADHD and non-ADHD participants would differ in attention to and usage of the non-instructed feature position. Less shielding of the instructed stimulus feature color should result in more attention to stimulus position. So potentially ADHD participants might show a larger RT difference between extreme and less extreme stimulus positions. Increased attention to the non-instructed feature can foster covariance learning involving the non-instructed feature (cf. Jiménez and Méndez, 1999; Hoffmann and Sebald, 2005) serving as a basis for usage of the non-instructed stimulus feature position for response selection. Accordingly, we expected ADHD participants to show a stronger increase in the percentage of ambiguous stimuli responded to in line with the color-position covariation across blocks of practice. Furthermore, stronger color usage should also show when instructed vs. learned stimulus feature are pitted against each other in deviant trials. Here, ADHD participants should show a higher percentage of responses in line with the learned rather than the instructed stimulus feature. Finally, stronger learning of the color-position covariation should lead to stronger response conflict when the covariation is broken in deviant trials. Resolution of a stronger conflict should take more time. Hence, we expected that the RT slowing in trials with deviant stimuli (with correct response) relative to standard stimuli should be larger in ADHD participants.

## Results

We first analyzed RTs and error rates in standard trials. This allowed to check whether ADHD and non-ADHD participants were able to perform the experimental task and whether there were indications that attention to the non-instructed feature position might differ between these groups. Afterward, we turned to ambiguous trials to check how usage of the covarying stimulus feature increased with practice when the instructed feature was not informative. Next, we analyzed the test phase where the instructed and the non-instructed feature were pitted against each other. Finally, we compared self-reports on noticing and using the alternative stimulus feature for participants with and without ADHD and explored how self-report was related to performance. With respect to ANOVAs, note that in case the Mauchly test indicated a violation of the sphericity assumption, we report unadjusted degrees of freedom together with the Greenhouse–Geisser correction factor ε – which we applied in determining the *p*-value.

### Performance in standard trials across blocks

**FIGURE 2:**
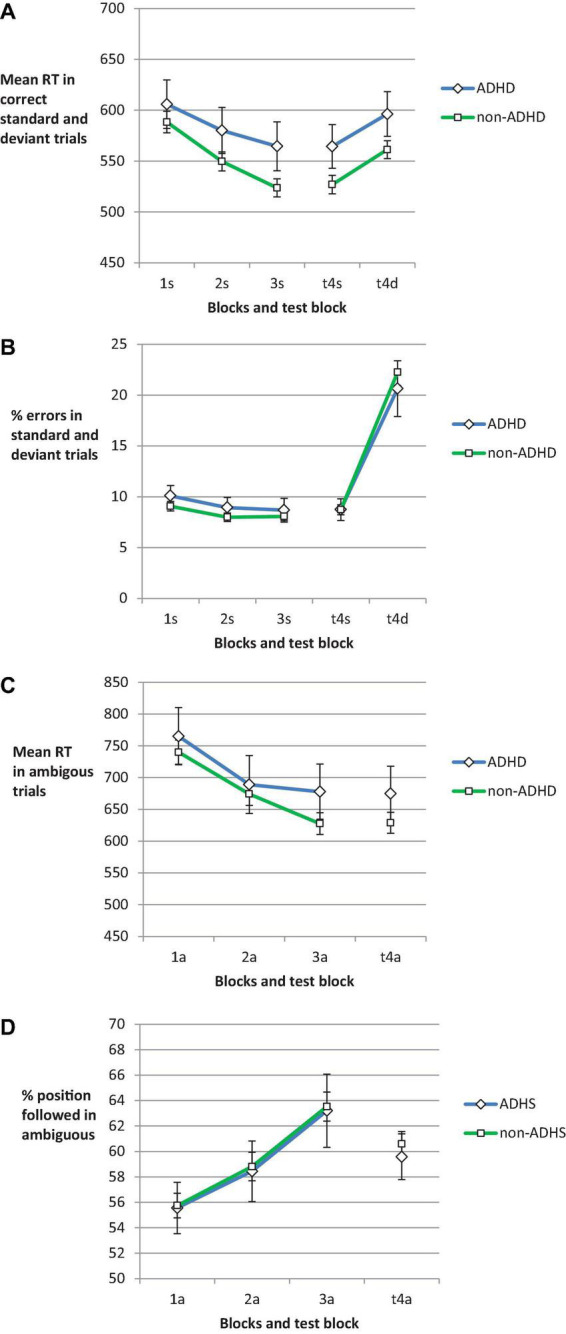
Mean RT **(A)** and error rate **(B)** in standard trials for Blocks 1, 2, and 3 (practice phase), and standard and deviant trials in Block 4 (test phase). Panel **(C)** shows the mean RT and panel **(D)** shows the proportion of responses in line with the color-position covariation in color-ambiguous trials. The error bars depict the between subjects SE of the mean. On the *x*-axis, numbers indicate the block. Letters indicate the trial type: s, standard; d, deviant; a, ambiguous.

Performance differences in standard trials between ADHD and non-ADHD participants early in practice could hint at differences in instruction implementation. This would lead to a different basis for a reduction of shielding of the instructed task later in practice. Yet, our analyses suggested that there were no such differences in performance in standard trials. Analyzing the mean RTs by a 2 (Group) × 3 (Block) mixed ANOVA for standard trials with correct response (Figure 2A) showed that there was only a main effect of Block, *F*(2,486) = 33.91, *p* < 0.001, $\eta_{p}^{2}=0.112$, ε = 0.771. There was neither a main effect of Group, *F*(1,243) = 1.78, *p* = 0.184, $\eta_{p}^{2}=0.007$, nor an interaction of Group and Block, *F*(2,486) = 1.62, *p* = 0.204, $\eta_{p}^{2}=0.007$, ε = 0.771. For the error rates (Figure 2B) there was a main effect of Block, *F*(2,486) = 3.82, *p* = 0.027, $\eta_{p}^{2}=0.015$, ε = 0.899, but neither a main effect of Group nor an interaction with Block (*F*s < 1).

In order to test whether participants were able to deal even with stimuli with small difference in color frequency, we analyzed RT in trials with correct response as well as error rate depending on color ratio. If, for instance, ADHD participants would show a particularly high error rate on hard-to-discriminate stimuli, this might on the one hand reduce their opportunities for covariation learning. On the other hand, they might experience this as a prompt to try to apply covariation knowledge acquired earlier (cf. Touron and Hertzog, 2004a,b; Haider et al., 2005). The ratio of cyan and magenta squares varied in standard trials between 10:2 and 2:10. The ratios that were the hardest to discriminate were 7:5 and 5:7. For this analysis, we used the difference between the dominant and the infrequent color as independent variable. So 10:2 and 2:10 yielded a difference of 8, while 7:5 and 5:7 yielded a difference of 2.

**FIGURE 3:**
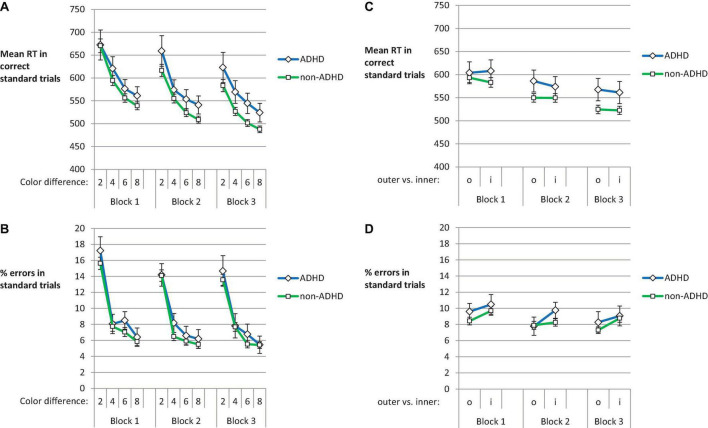
Mean RT **(A)** and error rate **(B)** in standard trials depending on color difference for Blocks 1, 2, and 3. Panels **(C,D)** show the mean RT and error rate for outer and inner stimulus positions (o and i for outer and inner position). The error bars depict the between subjects SE of the mean.

Figures 3A,B show the average RT and error data per block of practice and color difference for participants with and without ADHD. The 2 (Group) × 3 (Block) × 4 (Color Difference) mixed ANOVA on RT documented the already reported main effect of Block, *F*(2,486) = 34.48, *p* < 0.001, $\eta_{p}^{2}=0.124$, ε = 0.772. More interestingly, there was a main effect of Color Difference, *F*(3,729) = 142.89, *p* < 0.001, $\eta_{p}^{2}=0.37$, ε = 0.412, as RTs were substantially prolonged in trials with smaller compared to larger color difference. The interaction of Block and Color Difference, *F*(6,1458) = 4.31, *p* = 0.001, $\eta_{p}^{2}=0.017$, ε = 0.76, was qualified by a three-way interaction of Block, Color Difference and Group, *F*(6,1458) = 2.38, *p* = 0.027, $\eta_{p}^{2}=0.01$, ε = 0.76. In non-ADHD participants the impact of Color Difference on RT decreased from Block 1 to Block 3. ADHD participants instead showed this reduction after Block 2. The RT advantage of the largest compared to the smallest color difference for Block 1 to Block 3 was *M* = 131.33 ms, *M* = 107.58 ms and *M* = 95.74 ms in non-ADHD participants. In ADHD participants it was *M* = 111.33 ms, *M* = 118.2 ms, and *M* = 98.82 ms (for Blocks 1, 2, and 3, respectively). There were no other main effects or interactions (*F*s ≤ 1.74).

The corresponding ANOVA on% errors only revealed a main effect of Block, *F*(2,486) = 3.88, *p* = 0.021, $\eta_{p}^{2}=0.016$, ε = 0.898, and of Color Difference, *F*(3,729) = 169.29, *p* < 0.001, $\eta_{p}^{2}=0.04$, ε = 0.795. While error rates were substantially higher in trials with small as compared to large color difference, participants could correctly respond in most of the trials even in case of low color discriminability. There were no other main effects or interactions (*F*s ≤ 1.19).

In order to follow up on the hypothesized difference in the amount of attention to the non-instructed stimulus feature position, we analyzed the RTs to stimuli at extreme vs. medium position (Figures 3C,D) using a 2 (Group) × 3 (Block) × 2 (Position Extremeness) mixed ANOVA. We obtained a main effect of Position Extremeness, *F*(1,243) = 7.71, *p* = 0.006, $\eta_{p}^{2}=0.031$, reflecting that RTs were overall longer in trials with stimuli at the upper or lower border of the reference frame compared to upper and lower stimuli closer to the middle of the frame. More extreme positions lead to longer RTs. In line with the already reported decrease of RT with practice, there was a main effect of Block, *F*(2,486) = 34.03, *p* < 0.001, $\eta_{p}^{2}=0.123$, ε = 0.771. Figure 3C suggests that RTs developed differentially for ADHD and non-ADHD participants across practice for extreme and medium stimulus positions. This was confirmed by a significant three-way interaction of Block, Group, and Position Extremeness, *F*(2,486) = 5.55, *p* = 0.004, $\eta_{p}^{2}=0.022$, ε = 0.998. In ADHD participants, RTs to outer vs. inner positions began to differ with practice (*p* = 0.038, for the two-tailed *t*-test in Block 2). In non-ADHD participants this difference was present in Block 1 (*p* < 0.001), but ceased afterward. There was neither a main effect of Group nor any other interaction (*F*s ≤ 1.59).

In the corresponding ANOVA on error rates, there was a main effect of Position Extremeness, *F*(1,243) = 18.31, *p* < 0.001, $\eta_{p}^{2}=0.07$, reflecting that the reactions to stimuli at the less extreme positions were overall more error prone. Due to an overall reduction of error rate across practice, there was a main effect of Block, *F*(2,486) = 3.69, *p* = 0.026, $\eta_{p}^{2}=0.015$, ε = 0.922. Yet, there neither was a main effect of Group nor any interaction (*F*s ≤ 1.37).

### Ambiguous trials

In order to follow up on the hypothesis that ADHD participants might with practice show a stronger increase in usage of the non-instructed stimulus feature position for response selection, we analyzed the percentage of ambiguous trials with response in line with the color-position covariation. The rate increased across blocks of practice (Figure 2D). In the 2 (Group) × 3 (Block) mixed ANOVA, there was a main effect of Block, *F*(2,486) = 13.6, *p* < 0.001, $\eta_{p}^{2}=0.053$, but neither a main effect of Group nor an interaction with Block (*F*s < 1). *Post hoc* power analyses (Faul et al., 2007) suggested that by recruiting 43 ADHD participants we have reached decent power to detect a differential change across blocks. Comparing the change across blocks for 43 ADHD vs. 43 non-ADHD (i.e., orienting this estimate at the smaller sub-sample) participants would have yielded a power of 0.9 to detect an effect of $\eta_{p}^{2}=0.025$.

The RTs for ambiguous trials decreased with practice (Figure 2C). The corresponding ANOVA showed a main effect of Block, *F*(2,486) = 24.71, *p* < 0.001, $\eta_{p}^{2}=0.092$, ε = 0.858, but neither a main effect of Group nor an interaction with Block (*F*s < 1).

Interestingly, Figure 2D suggests that the introduction of deviant trials in the test block was accompanied by a reduction of position usage in ambiguous trials. There was a decrease in % position-following in ambiguous trials from the last practice block to the test block. We analyzed this with an ANOVA on percent position responses in ambiguous trials in Blocks 3 and 4 and Group. There was only a significant main effect of Block, *F*(1,243) = 5.86, *p* = 0.016, $\eta_{p}^{2}=0.024$. There was neither a main effect of Group nor an interaction (*F*s < 1).

### Deviant trials

Investigating the hypothesized group differences in resolution of response conflict, we evaluated deviant slowing. We compared the RT of standard trials with correct responses in Block 4 and deviant trials (Figure 2A) in which participants responded in line with the instructed stimulus feature color (rather than the conflicting feature position). In the Group by Trial Type ANOVA there was a main effect of Trial Type, *F*(1,243) = 46.46, *p* < 0.001, $\eta_{p}^{2}=0.16$, showing that participants responded more slowly to the deviant trials than to the standard trials. There was neither a significant main effect of Group, *F*(1,243) = 2.91, *p* = 0.089, $\eta_{p}^{2}=0.012$, nor an interaction (*F* < 1).

In order to evaluate response selection in deviant trials, error rate in standard trials in Block 4 was used as a baseline (Figure 2B). Accordingly, we repeated the above ANOVA taking the percentage of trials with instruction-incongruent response (i.e., error in standard trial and position-followed response in deviant trial) as dependent variable. In the Group by Trial Type ANOVA there was a main effect of Trial Type, *F*(1,243) = 74.58, *p* < 0.001, $\eta_{p}^{2}=0.235$. Error rates were higher in deviant trials. There was neither a significant main effect of Group nor an interaction (*F*s < 1).

### Self-report and relation to performance

**FIGURE 4:**
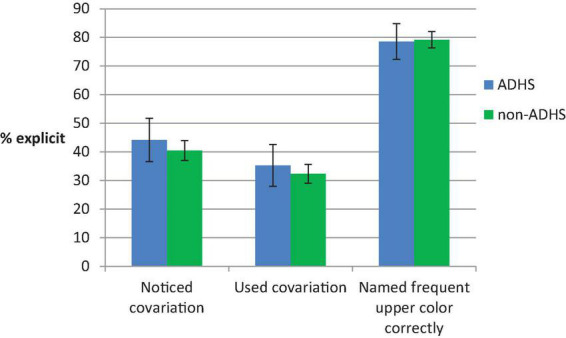
Self-report indicators of explicit knowledge and usage of the color-position covariation. The error bars depict the between subjects SE of the proportion.

In all three self-report measures, the proportion of participants indicating explicit knowledge about the color-position-covariation did not differ between the ADHD and non-ADHD group (Chi-square tests indicating *p*s ≥ 0.211). As shown in Figure 4, the proportion of participants who could correctly assign that magenta was the color which was most frequent for upper stimuli was higher than the proportion of participants reporting to have noticed and/or used the color-position covariation. Yet, while for the former question the guessing baseline is 50%, we have no data for a guessing baseline for the other two questions.

**FIGURE 5:**
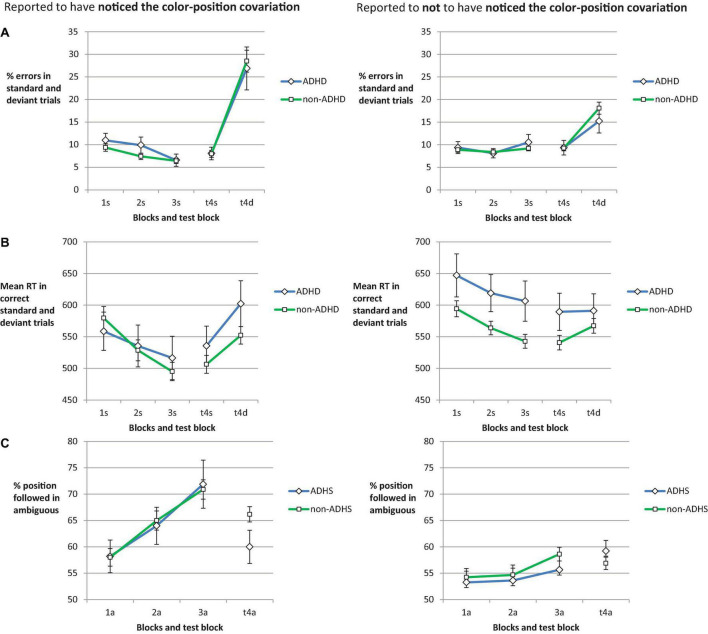
For participants reporting to have noticed the color-position covariation (left column) vs. not have noticed the covariation (right column) error rate **(A)** and mean RT **(B)** are shown for standard trials for Blocks 1, 2, and 3 (practice phase), and standard and deviant trials in Block 4 (test phase). Panel **(C)** shows the proportion of responses in line with the color-position covariation in color-ambiguous trials. The error bars depict the between subjects SE of the mean. On the *x*-axis, numbers indicate the block. Letters indicate the trial type: s, standard; d, deviant; a, ambiguous.

In a similar covariation learning task as in our study, Schuck et al. (2015) found that only participants who reported to have noticed the color-position covariation showed behavioral and fMRI indicators of strategy change. Touron and Hertzog (2004a,b) showed that groups with similar levels of knowledge about redundancies in the task material can differ in the extent to which this knowledge is actually applied. In their studies older as compared to younger adults were hesitant to apply a shortcut strategy. Hence, we were interested to explore to what extent strategy change depended on awareness and whether the link between strategy change and awareness differed for ADHD and non-ADHD participants. As in the above analyses we used response choice in ambiguous and deviant trials as measures of strategy change and in addition checked response slowing in deviant trials. In these explorative analyses we differentiated between participants who reported to have noticed vs. reported to not have noticed the covariation (see Figure 5). A subsample of *n* = 81 (40.1%) non-ADHD participants reported to have noticed the color position-covariation, as opposed to *n* = 121 who did not. In the ADHD group, *n* = 19 (44.2%) reported awareness, as opposed to *n* = 24 who did not.

#### Errors and slowing on deviant trials in the test block

Analyzing the error rate of standard and deviant trials from Block 4 (Figure 5A) in a 2 (Awareness) × 2 (Group) × 2 (Trial Type) mixed ANOVA revealed a main effect of Awareness, *F*(1,241) = 8.11, *p* = 0.005, $\eta_{p}^{2}=0.033$, as error rate was higher in aware as compared to unaware participants. The increase in error rate in deviant as compared to standard trials was reflected in a main effect of Trial Type, *F*(1,241) = 96.11, *p* < 0.001, $\eta_{p}^{2}=0.285$. As this increase was more pronounced in aware participants, there was an interaction of Trial Type and Awareness, *F*(1,241) = 22.32, *p* < 0.001, $\eta_{p}^{2}=0.085$ (other *F*s < 1). Follow-up analyses showed that not only aware ADHS and non-ADHS participants (*p* < 0.001 in both paired *t*-tests) showed a significant increase in error rate but also unaware ADHS participants [*t*(23) = 2.47, *p* = 0.011] and non-ADHS participants (*p* < 0.001).

The corresponding ANOVA on RT (Figure 5B) showed a main effect of Trial Type, *F*(1,241) = 58.62, *p* < 0.001, $\eta_{p}^{2}=0.196$, as deviant trials led to longer RTs as compared to standard trials. The interaction of Trial Type and Awareness, *F*(1,241) = 21.92, *p* < 0.001, $\eta_{p}^{2}=0.083$, suggested that deviant slowing was more pronounced in aware as compared to unaware participants. The triple interaction of Trial Type, Group, and Awareness, *F*(1,241) = 6.97, *p* = 0.009, $\eta_{p}^{2}=0.028$, reflected that non-aware ADHD participants did not seem to show deviant slowing. There was no significant main effect of awareness, *F*(1,241) = 3.17, *p* = 0.076, $\eta_{p}^{2}=0.013$ (other *F*s < 1). Follow-up analyses indicated that there was no deviant slowing in unaware ADHS participants, *t*(23) = 0.101, *p* = 0.921, while deviant trials led to longer RTs as compared to standard trials in all other three subgroups (*p*s < 0.001).

With respect to the above group average effects, two scenarios can be differentiated. On the one hand it is possible that those participants who strongly used position were also the ones who (in some trials) managed to resolve the strong response conflict, leading to a slowed but correct response. This should be reflected in a positive correlation between RT costs and error costs of deviants. On the other hand, it is possible that strong position users hardly ever resolved response conflicts, because position was dominating response selection. This should be reflected in a negative correlation. Results supported the former view. We observed a positive Spearman rank correlation in non-ADHS participants who had noticed the covariation (*r* = 0.249, *p* = 0.025) and those unaware of the covariation (*r* = 0.185, *p* = 0.042). There were no significant correlations for aware or unaware ADHS participants (*p*s ≥ 0.174).

#### Ambiguous trials across blocks of practice

In ambiguous trials, the percentage of responses in line with the covariation (Figure 5C) was related to Awareness as well. The 2 (Awareness) × 2 (Group) × 3 (Block) mixed ANOVA showed that there was a main effect of awareness, *F*(1,241) = 31.11, *p* < 0.001, $\eta_{p}^{2}=0.114$, and of Block, *F*(2,482) = 15.94, *p* < 0.001, $\eta_{p}^{2}=0.062$, qualified by an interaction of Block and Awareness, *F*(2,482) = 3.91, *p* = 0.021, $\eta_{p}^{2}=0.016$ (other *F*s < 1). Only aware participants strongly used the covarying stimulus feature for response selection and showed a marked increase in doing so across blocks of practice.

## Discussion

Flexibility of attention has been suggested as a beneficial characteristic of people affected by ADHD (cf. Hoogman et al., 2020). Here, we explored whether this flexibility might provide an advantage in overcoming the shielding of the task structure implemented based on instructions (cf. Dreisbach and Haider, 2008) and instead allow to attend and use an alternative stimulus feature not part of the instruction. To this end we developed an incidental covariation learning task that was similar to the situation at the traffic light: while being instructed to attend and use color for determining the appropriate response, people could also take into account whether the stimulus position was high or low, as this was correlated with stimulus color.

We observed no differences in behavioral and self-report indicators of usage of the covarying stimulus feature position. With practice, both groups increased the rate of responses in line with the color-position covariation in ambiguous trials (in which the instructed stimulus feature could not be discriminated) to the same extent. Also when instructed and learned feature were put into conflict in the test phase, groups showed an identical impact of the non-instructed stimulus feature. The amount of slowing of reactions when the covariation was broken did not differ among groups, suggesting that strength and resolution of conflict between opposing response tendencies (cf. Botvinick et al., 2001) were similar for participants with and without ADHD. Both groups showed a strong increase in error rate in deviant trials (color-position covariation broken) compared to standard trials (color-position covariation obeyed). This increase in error rate implies that the learned stimulus feature could at least in some trials override the instructed stimulus feature color. Yet, there was neither a group difference in these deviant errors nor in potential reactive effects of introducing deviant trials. In particular, the proportion of position-based responses in ambiguous trials decreased from the last practice block to the test block which introduced deviant trials. One can suspect that participants to some extent reduced their reliance on the non-instructed feature as a reaction to deviant trials. Earlier work has shown that practice can lead to a situation where a potential response conflict remains undetected and unresolved. Evidence for this would be a high rate of fast erroneous responses when a set introduced earlier is no longer valid (cf. Woltz et al., 2000). Our results suggest that the risk that practice with task material with redundant features makes participants blind to response conflict once it occurs did not differ among groups. Taken together, our results suggest that ADHD individuals show equal likelihood of overcoming an instructed task set and employing a shortcut instead. This is in line with work suggesting that individuals with ADHD can obtain equal or even better outcomes compared to non-ADHD persons for some task demands (Greven et al., 2018; Lesch, 2018).

On the one hand, self-report and behavioral measures of strategy change showed no group differences. On the other hand, we not only assessed strategy change, but also employed a measure that should tap allocation of intention to the covarying stimulus feature. This is a potential prerequisite of strategy change (cf. Frensch and Miner, 1994; Jiménez and Méndez, 1999; Hoffmann and Sebald, 2005; Gaschler et al., 2012). Our measure of attention allocated to the stimulus position provided a hint, that ADHD participants were more strongly attending the non-instructed stimulus feature. With practice, their RT was increasingly influenced by whether the stimulus was at an extreme vs. less extreme position. Following up on why more attention to position did not result in more strategy change in ADHD participants, we refer to the self-report measures of awareness. Participants from both groups were equally likely to report to have noticed the covariation between color and position. There is thus no basis to assume that groups might have differed in reluctance to apply a shortcut strategy once this option was noticed. Further work should instead focus on detailing why potential differences in attention do not lead to differences in awareness.

Given that we obtained no group difference in awareness, it is relevant to consider evidence for that our awareness measure was valid enough to capture aspects of strategy change. Exploratory analyses targeted to what extent becoming aware of the color-position covariation accounted for the variance in strategy change. The simple (and potentially suggestive) binary self-report measure of awareness used in the current study seemed to indeed account for a large share in the variance of strategy change measures. First and foremost, those participants who reported that they had noticed the color-position covariation were the ones who with practice started to use the non-instructed stimulus feature position for response selection in ambiguous trials (and also in deviant trials). We assume that this is indicative of a strategy change. Schuck et al. (2015) suggest that when covariation learning has accumulated knowledge about a potential alternative way to perform the task (i.e., by using the covarying feature instead of the instructed stimulus feature), people engage in a decision process on whether to stick to the instructed way of performing the task or to change the strategy. Some participants notice the covariation, but decide not to use it. Gaschler et al. (2019), see also Gaschler et al. (2015); Haider and Frensch (2002), and Lustig et al. (2021) have reported further behavioral evidence for that the usage of the covarying stimulus feature takes place based on a top-down decision to change the task strategy. Using four colors and four positions Gaschler et al. (2019) could vary the frequency with which particular color-position pairings occurred during practice. In line with the view that participants decide to use the covarying stimulus feature in general, they observed that strategy change transferred from frequent to less frequent feature combinations. Furthermore, Gaschler et al. (2014) reported transfer of shortcut usage among tasks. These tasks only had in common *that* there was a redundancy in the task material which could be exploited for more efficient task processing based on a shortcut. Stimuli, responses, and shortcut did not overlap among the tasks. Apparently participants engage in a decision process on whether or not they use a shortcut option they have discovered.

In the current study, aware participants in many deviant trials used position instead of color for response selection. For unaware participants, this effect was present on a reduced level. A similar pattern was observed with respect to slowing in deviant trials. Aware participants showed stronger slowing when responding to deviant stimuli in line with the instructions. For unaware non-ADHD participants this slowing was reduced and for unaware ADHD participants slowing seemed absent. These results suggest that awareness of the color-position covariation was coupled to more strongly using the non-instructed stimulus feature position for response selection. On the one hand, weighting the feature position strongly in response selection (cf. Memelink and Hommel, 2013), led to errors on some deviant trials. On the other hand, on some trials aware participants seemed to have succeeded in resolving the response conflict (cf. Botvinick et al., 2001) between the opposing responses tendencies by the instructed and the learned stimulus feature. This might have led to the prolonged RTs in deviant trials with correct response. For unaware participants the influence of position on response selection was reduced. Placing strong weight on the instructed feature should have limited the response conflict to be resolved. This might explain the reduced deviant slowing. Given that all subgroups showed an effect of deviant trials in the error rate, knowledge about the color-position covariation apparently was acquired in all subgroups of participants and was influencing response selection. Without further empirical evidence we therefore are hesitant to interpret that non-aware ADHD participants apparently showed no deviant slowing.

The above results await replication and further exploration with larger samples of ADHD- and non-ADHD participants. The task used in the current study seems to be well suited for such a line of work. Our analyses showed that participants of either group can cope with the instructed task equally well. RT and error rates in standard trials were similar at the beginning of practice. This suggests that implementation of the instructed task set was similar among ADHD and non-ADHD participants. Therefore, the task seems to be suited to study potential differences in task shielding that might manifest with practice. The task seems suitable to study learning-based deviations from instructed task processing in diverse samples online. With our task, participant groups which might be difficult to recruit in lab-based studies can be tested online.

In order to evaluate the specifics and strengths of the current paradigm, it is interesting to discuss the overlap with work on the set effect in problem solving. For instance, in the Luchins water jar problem (Luchins, 1942), participants are to solve a series of mental calculation problems involving numbers for three jars and a number for the target jug. In the first problems the solution always requires the same mental operations in the same order among the three numbers presented in each trial. Participants then stick to the established way of processing even when later there are problems that could in principle be solved in a much more straightforward way, too. Afterward, problems are being presented, to which the established routine cannot be applied. Participants who had been exposed to the trials establishing the set take longer to find the solution to these problems or fail to find it at all. Work on the Einstellung effect with the water jar task has documented that participants are – despite searching for an alternative solution, strongly influenced by the activation of the elements of the known solution. For instance, think-aloud protocols in Blech et al. (2020) show that participants keep starting with the wrong element. Recent eyetracking work suggests that attention remains allocated to elements involved in the standard solution in those participants who do not eventually shift to the alternative strategy (Ninomiya et al., 2022). In a similar vein, eyetracking in chess problems has shown that a known solution captures attention even though problem solvers believe that they are occupied with an alternative solution approach (Bilaliæ et al., 2008). Apparently, people face difficulties directing attention away from the elements of the known but no longer applicable solution.

One obvious difference between work on the set effect and on practice-based deviation from the instructed task sets is that in work on the Einstellung effect the set is traditionally established unobtrusively *via* practice rather than by directly instructing people. This seems to point toward a void in research rather than to a structural difference. On the one hand, a set might in principle also be established by instruction. Thus, future work might compare, on the one hand, the stickiness of practice-induced vs. instruction-induced sets in problem solving tasks such as the water jar task. On the other hand, in incidental covariation learning tasks such as applied in the current work, the extent of shielding protecting an instructed task set vs. one found by trial and error might be compared experimentally. Recently, Navarre et al. (2022) have analyzed whether the anchoring effect and the Einstellung effect might overlap conceptually and with respect to responsible mechanisms. In either case activation of an element in working memory biases processing toward overlapping features and content.

The current incidental covariation learning task differs from problem solving tasks used to study the set effect in that our task is rather easy and broadly applicable. While with water jar problems (cf. Luchins and Luchins, 1994; Blech et al., 2020; Ninomiya et al., 2022) or chess problems (cf. Bilaliæ et al., 2008; Sheridan and Reingold, 2013) participants need to be selected such that their skills are sufficient to perform the basic tasks (mental calculation or playing chess at a decent level), our covariation learning task is more broadly applicable. The current work shows that our task is suitable for participants with and without ADHD. A goal for future studies can be to compare children, adolescents and adults with and without ADHD. This seems promising as Luo et al. (2019) have summarized that reduction of ADHD symptoms over development correlates with the maturation of the prefrontal cortex and related circuitry. Based on reasoning about this maturation, Schuck et al. (2022) have recently applied a covariation learning task to compare children and young adolescents. Strategy change was shown by 27.5% of the 8–10 year olds and by 28.2% of the adults. In their setup, position was the instructed stimulus feature and color the seemingly irrelevant feature. Despite this difference, their results suggest that the current paradigm can be applied in different age cohorts with and without ADHD. Notably, some features of the paradigm seem particularly useful for such research. Employing proportion of responses in ambiguous and deviant trials as dependent variable might bring about advantages in comparing populations which might strongly differ in RTs. With RT-based measures one would have to decide among the options of (1) using simple RT differences or (2) proportional slowing (to compensate for differences in general speed). Furthermore, different from RT-based measures, outliers are less likely to occur when using percent of responses.

Hoogman et al. (2020) summarize that the idea has gained prominence that ADHD can be beneficial for creative thought by fostering divergent flexible attention and thinking. They suggest that the basis for the many inconsistent findings on the issue (cf., White and Shah, 2006, 2016; Paek et al., 2016) can be attributed to the divergence of research designs, samples, and creativity tasks. With the current work we provide a first step toward addressing this issue by using a task that can (a) be used in diverse populations even in an online setting, (b) is simple to instruct and robust in handling and can (c) offer detailed converging behavioral indicators of attention to and employment of non-instructed stimulus-features. The latter allows to trace the process of deviation from a standard way of task performance and relate it to self-report indicators. Given that the current study established that participants with and without ADHD can handle the covariation learning task well, difficulty of the instructed task can be increased further in future studies in order to increase the potential gain by using the non-instructed feature for response selection. Yet, it has to be considered that noticing an alternative stimulus feature vs. using it might be fostered by opposing conditions. Shifting attention away from the features of the instructed task set might be especially likely when difficulty is very *low*. For instance, Stawarczyk et al. (2011) and Thomson et al. (2014) reported that shifts from task-related to task-unrelated thoughts are observed during low cognitive task demands (cf. Bozhilova et al., 2018). To balance the different impact that low vs. high task difficulty might have on noticing vs. applying an alternative stimulus feature, trials with high and with low difficulty might be combined. For instance, a schedule with changes in task difficulty might be specifically suited to allow individuals with ADHD to play out potential advantages in flexible attention shifting. During low difficulty phases they might be more likely to start attending task-unrelated features compared to other people. During phases of high difficulty, the knowledge gained by reducing task shielding and attending non-instructed features might be employed to support task performance (cf., Touron and Hertzog, 2004a,b; Haider et al., 2005).

## Data availability statement

The datasets presented in this study can be found in online repositories. The names of the repository/repositories and accession number(s) can be found below: https://osf.io/ab46j/.

## Ethics statement

The studies involving human participants were reviewed and approved by Ethics Committee of the Department of Psychology of Humboldt-Universität, Berlin. The participants provided their written informed consent to participate in this study.

## Author contributions

MK contributed the lab.js code for running the experiment. RG and BD-K contributed the data analysis and the primary draft of the manuscript. All authors contributed to designing and planning the research and the write-up of the manuscript.
